# LncRNA *ZNF503-AS1* promotes RPE differentiation by downregulating *ZNF503* expression

**DOI:** 10.1038/cddis.2017.382

**Published:** 2017-09-07

**Authors:** Xue Chen, Chao Jiang, Bing Qin, Guohua Liu, Jiangdong Ji, Xiantao Sun, Min Xu, Sijia Ding, Meidong Zhu, Guofu Huang, Biao Yan, Chen Zhao

**Affiliations:** 1 Department of Ophthalmology, The First Affiliated Hospital of Nanjing Medical University, State Key Laboratory of Reproductive Medicine, Nanjing 210029, China; 2 Department of Ophthalmology and Vision Science, Eye & ENT Hospital, Shanghai Medical College, Fudan University, Shanghai 200023, China; 3 Key Laboratory of Myopia of State Health Ministry (Fudan University) and Shanghai Key Laboratory of Visual Impairment and Restoration, Shanghai 200023, China; 4 Department of Ophthalmology, The First People’s Hospital of Suqian, Suqian 223800, China; 5 Department of Ophthalmology, Qilu Children’s Hospital of Shandong University, Jinan 250000, China; 6 Department of Ophthalmology, Children’s Hospital of Zhengzhou, Zhengzhou 450053, China; 7 Department of Ophthalmology, Northern Jiangsu People’s Hospital, Yangzhou 225000, China; 8 Save Sight Institute, Discipline of Clinical Ophthalmology and Eye Health, The University of Sydney, Sydney, NSW 2000, Australia; 9 Department of Ophthalmology, The Third Affiliated Hospital of Nanchang University, Nanchang 330000, China; 10 Research Center, Eye & ENT Hospital, Shanghai Medical College, Fudan University, Shanghai 200023, China

## Abstract

Long noncoding RNAs (lncRNAs) have important roles in various biological processes. Our previous work has revealed that dedifferentiation of retinal pigment epithelium (RPE) cells contributes to the pathology of age-related macular degeneration (AMD). Herein, we show roles of lncRNAs in RPE differentiation. We used microarray to identify lncRNA expression profiles in human induced pluripotent stem cells (hiPSCs) and hiPSC-derived RPE cells. A total of 217 differentially expressed lncRNAs along with the differentiation were initially identified, among which 13 lncRNAs showed a consistent fold change of over 2. LncRNA *ZNF503-AS1*, located in the cytoplasm of RPE cells, was found consistently upregulated along with RPE differentiation, and downregulated in the RPE-choroid of AMD patients. *In vitro* study further suggested that *ZNF503-AS1* insufficiency could inhibit RPE differentiation, and promote its proliferation and migration. As *ZNF503-AS1* is transcribed from the antisense strand of the *ZNF503* gene locus, we further revealed its regulatory role in *ZNF503* expression. *ZNF503-AS1* was reversely correlated with *ZNF503* expression. Our results also suggested that *ZNF503* could inhibit RPE differentiation, and promote its proliferation and migration. Thus, *ZNF503-AS1* potentially promotes RPE differentiation through downregulation of *ZNF503* expression. In addition, nuclear factor-*κ*B was recognized as a potential upstream transcript factor for *ZNF503-AS1*, which might participate in promoting RPE differentiation by regulating the expression of *ZNF503-AS1*. Taken together, our study identifies a group of RPE differentiation relevant lncRNAs, and the potential role of *ZNF503-AS1* in the pathology of atrophic AMD, which might help with the intervention of AMD patients.

Retinal pigment epithelium (RPE) is a monolayer of cuboidal, polarized, and pigmented epithelial cells located in the outer retina between photoreceptors and choroidal vessels.^1, 2, 3^ RPE forms a part of the blood/retina barrier, secrets multiple growth factors, and is crucial in maintaining regular retinal functions.^2, 3, 4^ Dysfunction and depletion of RPE cells are involved in multiple retinal degenerations, including age-related macular degeneration (AMD).^2, 5, 6, 7^ AMD is a universal leading cause for irreversible vision loss in people aged over 55.^8, 9, 10^ Clinically, AMD can be classified into two major categories, namely atrophic and exudative AMD.^10^ Our group has previously recognized RPE dedifferentiation as a crucial contribution factor to the pathogenesis of atrophic AMD.^1, 3^ Atrophic AMD is typified by subepithelial deposits and degeneration of RPE cells involving but not limited to the macular region.^10^ No efficient therapies have been developed for atrophic AMD. Therefore, better insights into its pathology and seeking for a putative therapeutic target have become the focus of current researches. On the basis of our previous findings, inhibiting RPE dedifferentiation may retard or block AMD disease course, thus helping with better control of AMD patients.

Long noncoding RNAs (lncRNAs) are transcripts longer than 200 nucleotides structurally homologous to protein-coding mRNAs, but has little or no protein-coding potential.^11^ LncRNAs can modulate gene expressions as competing endogenous RNAs (ceRNAs).^11, 12, 13, 14^ The ceRNA hypothesis defines a microRNA (miRNA)-mediated post-transcriptional regulatory network. In this network, protein-coding and non-protein-coding RNAs share one or more miRNA response elements and compete for miRNA binding, further modulating each other’s expressions.^12, 15^ LncRNAs have been reported to have regulatory roles in diverse biological processes, including cell differentiation, stem cell maintenance, and epigenetic regulation.^16, 17, 18^ Dysregulation of lncRNAs is found involved in multiple human diseases such as cancer, neurological problems, and cardiovascular diseases.^19^ Roles of lncRNAs in ocular diseases, such as diabetic retinopathy, proliferative vitreoretinopathy, glaucoma, ocular tumors, and ocular neovascularization, have also been identified.^11, 14, 20, 21, 22, 23, 24, 25, 26^ However, the association between lncRNAs and RPE differentiation is poorly understood. In this study, we aim to reveal the roles of a lncRNA, *ZNF503-AS1*, in RPE differentiation, and to seek for a lncRNA-based potential therapeutic target for dry AMD.

## Results

### Differently expressed lncRNAs during RPE differentiation

**Figure 1:**
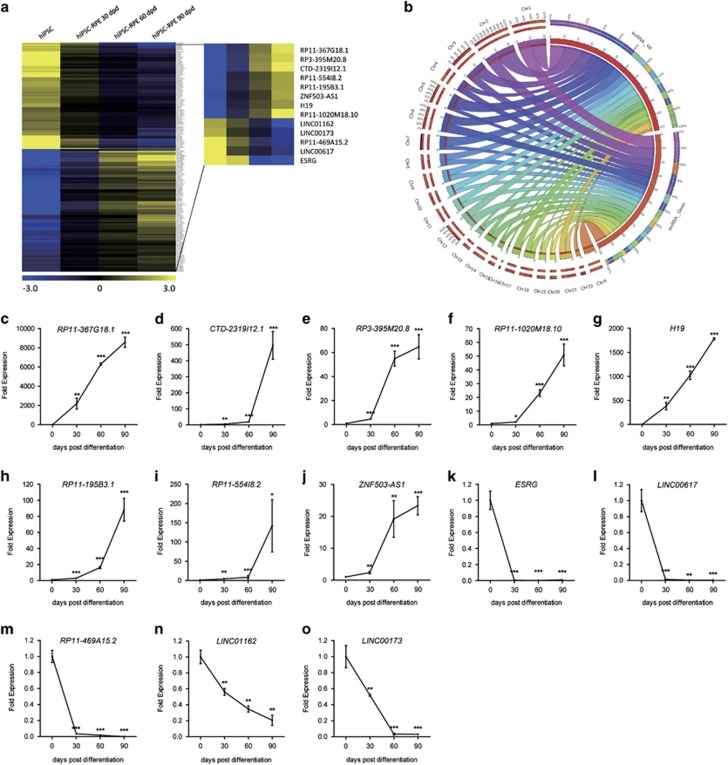
Expression profiles of lncRNAs in RPE differentiation. (**a**) Hierarchical clustering of all 217 lncRNAs with a consistent expressional change along with the differentiation. Thirteen lncRNAs with consistent fold change of over 2 are highlighted. Blue: downregulated lncRNAs; yellow: upregulated lncRNAs. (**b**) Chromosomal locations of the 217 differentially expressed lncRNAs are shown as a Circos plot. (**c**–**o**) The 13 lncRNAs with consistent fold change of over 2 are selected for validation of the microarray results in hiPSC-RPE at 0, 30, 60, and 90 d.p.d., including upregulated *RP11-367G18.1* (**c**), *CTD-2319I12.1* (**d**), *RP3-395M20.8* (**e**), *RP11-1020M18.10* (**f**), *H19* (**g**), *RP11-195B3.1* (**h**), *RP11-554I8.2* (**i**), and *ZNF503-AS1* (**j**), and downregulated *ESRG* (**k**), *LINC00617* (**l**), *RP11-469A15.2* (**m**), *LINC01162* (**n**), and *LINC00173* (**o**)

To reveal the roles of lncRNAs on RPE differentiation, we used microarrays to modulate the expression profiles of lncRNAs and mRNAs in human induced pluripotent stem cells (hiPSCs) and in hiPSC-derived RPE cells (hiPSC-RPE) at 30, 60, and 90 days post differentiation (d.p.d.), respectively. Pluripotency of the hiPSC colonies and competence of differentiation have been previously determined.^3^ Among all identified lncRNAs, only those recorded in Encyclopedia of DNA Elements (ENCODE) showed a consistent expressional change, and presented a fold change of over 2 in the 30 d.p.d. hiPSC-RPE compared to undifferentiated hiPSC were included. A total of 217 differentially expressed lncRNAs, including 116 upregulated and 101 downregulated lncRNAs, were sorted out with their chromosomal locations annotated (Figures 1a and b; Supplementary Table S1). Of all 217 identified lncRNAs, 13 showed a consistent fold change of over 2 among all time points along with the differentiation, indicating their crucial roles in RPE differentiation, and were selected for further analysis (Table 1; Figure 1a).

To validate the microarray data, we next confirmed expressions of the 13 lncRNAs using real-time PCR. Agreed with the microarray data, lncRNAs *RP11-367G18.1* (ENST00000452675), *CTD-2319I12.1* (ENST00000590346), *RP3-395M20.8* (ENST00000416860), *RP11-1020M18.10* (ENST00000548135), *H19* (ENST00000414790), *RP11-195B3.1* (ENST00000436340), *RP11-554I8.2* (ENST00000417112), and *ZNF503-AS1* were found consistently upregulated (Figures 1c–j), while *ESRG* (ENST00000583516), *LINC00617* (ENST00000503525), *RP11-469A15.2* (ENST00000427825), *LINC01162* (ENST00000447262), and *LINC00173* (ENST00000480237) were downregulated (Figures 1k–o).

### *ZNF503-AS1* is mainly localized in cytoplasm

**Figure 2:**
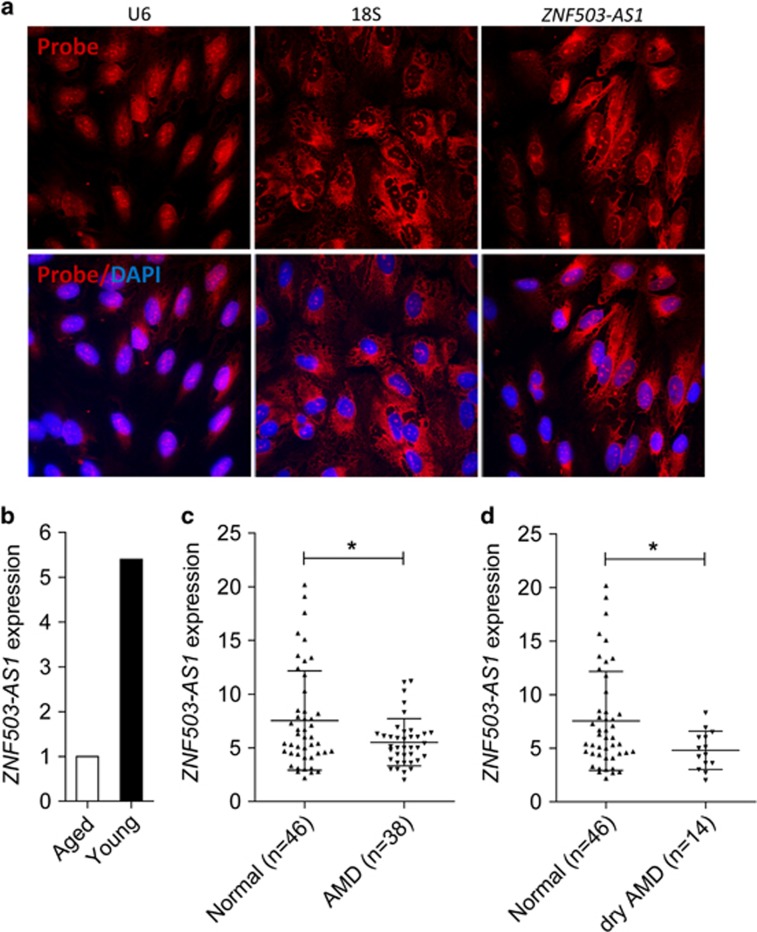
Intracellular localization and clinical relevance of *ZNF503-AS1*. (**a**) RNA-FISH suggested that *ZNF503-AS1* was mainly localized in the cytoplasm of ARPE19 cells. U6 was taken as a representative for nuclear localization, and 18S for cytoplasmic localization. (**b**) Relative expression of *ZNF503-AS1* in the macular RPE of an aged donor compared to a young donor. (**c** and **d**) *ZNF503-AS1* expressions compared between the extramacular RPE-choroid tissue of the control group and AMD patients (**c**) and dry AMD patients (**d**), respectively

*ZNF503-AS1* is an intergenic lncRNA located on chromosome 10q22.2, and is partly conserved among multiple species (Supplementary Figure S1). However, the biological function of *ZNF503-AS1* is largely unknown. Our above results confirmed that *ZNF503-AS1* was consistently upregulated along with the differentiation from hiPSC to RPE (Figure 1j). RNA-fluorescent *in situ* hybridization (FISH) suggested that *ZNF503-AS1* was expressed in human RPE (ARPE19) cells, and was mainly localized in cytoplasm (Figure 2a).

### Clinical relevance of *ZNF503-AS1*

Taken that RPE dedifferentiation was involved in the pathogenesis of retinal degenerative diseases,^1, 3^ we assumed that *ZNF503-AS1* expression would be reduced in dysfunctional RPE. To test our hypothesis, we compared *ZNF503-AS1* expression between macular RPE-choroid of a 70-year-old male and a 30-year-old female donor. Our previous study suggested that the male donor had RPE dysfunction.^3^ As expected, *ZNF503-AS1* expression was significantly reduced in the aged donor with RPE dysfunction when compared with the young female donor (Figure 2b). To further tell the role of *ZNF503-AS1* in AMD pathogenesis, we compared its expression among RPE-choroid samples of 38 AMD patients and 46 healthy controls. Personal particulars and clinical diagnosis for each participant were detailed before.^27^ As indicated in Figure 2c, *ZNF503-AS1* was downregulated in AMD patients when compared with controls. Subgroup analysis was then performed to see with which AMD subtype *ZNF503-AS1* was mostly correlated. AMD diagnostic criterion was described previously.^27^ Our findings suggested that *ZNF503-AS1* expression was only found reduced in RPE-choroid of dry AMD patients (*n*=16), a branch of atrophic AMD (Figure 2d). Taken together, our findings suggested that dysregulation of *ZNF503-AS1* is involved in RPE dysfunction, especially in atrophic AMD.

### *ZNF503-AS1* promotes RPE differentiation

**Figure 3:**
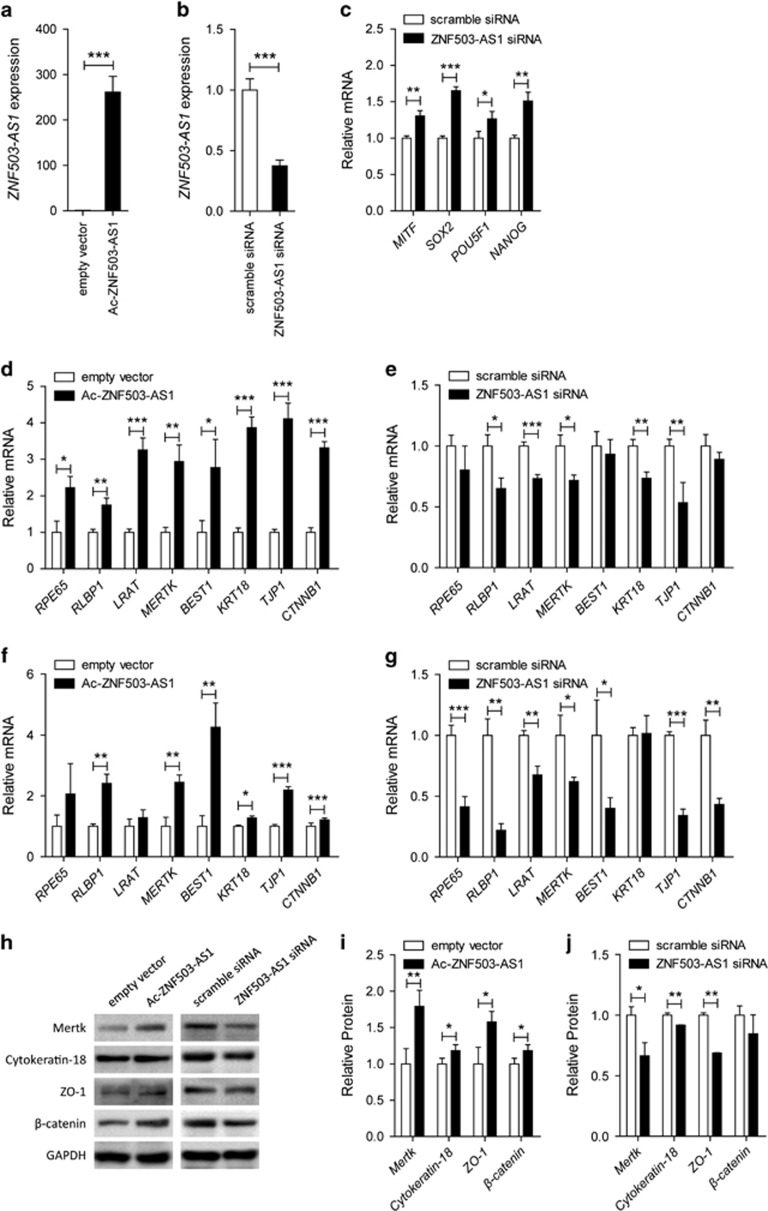
*ZNF503-AS1* promotes RPE differentiation. (**a** and **b**) Relative miRNA expression of *ZNF503-AS1* in hiPSC-RPE at 30 d.p.d. transfected with Ac-*ZNF503-AS1* plasmid compared to empty vector (**a**), and in cells transfected with *ZNF503-AS1* siRNA compared to scramble siRNA (**b**). (**c**) Relative mRNA expressions of RPE dedifferentiation-related markers, including *MITF*, *SOX2*, *POU5F1*, and *NANOG*, in hiPSC-RPE at 30 d.p.d. transfected with *ZNF503-AS1* siRNA compared to scramble siRNA. (**d**–**g**) Relative mRNA expressions of *RPE65, RLBP1*, *LRAT*, *MERTK*, *BEST1*, *KRT18*, *TJP1*, and *CTNNB1* in hiPSC-RPE at 30 d.p.d. (**d**) and ARPE19 cells (**e**) transfected with Ac-*ZNF503-AS1* compared with empty vector, and in hiPSC-RPE at 30 d.p.d. (**f**) and ARPE19 cells (**g**) transfected with *ZNF503-AS1* siRNA compared to scramble siRNA. (**h**–**j**) Relative protein expressions of Mertk, cytokeratin-18, ZO-1, and *β*-catenin in ARPE19 cells transfected with Ac-*ZNF503-AS1* compared with empty vector (**h** and **i**), and in cells transfected with *ZNF503-AS1* siRNA compared to scramble siRNA (**h** and **j**)

We next used transfection assays to modulate expression levels of *ZNF503-AS1* in cells to evaluate its role in RPE differentiation. Exogenous *ZNF503-AS1* was significantly upregulated in cells transfected with Ac-*ZNF503-AS1* plasmid (Figure 3a), and endogenous *ZNF503-AS1* was downregulated in cells transfected with *ZNF503-AS1* scramble small interfering RNA (siRNA; Figure 3b). Noteworthy, three pairs of siRNAs oligos targeting different regions of *ZNF503-AS1* were initially designed and tested, and the siRNA showing best efficiency and stability was chosen for further studies (Supplementary Figure S2A). Increased expressions of RPE dedifferentiation-related markers, including microphthalmia-associated transcription factor (*MITF*; NM_198159), *SOX2* (NM_003106), POU domain class 5 transcription factor 1 (*POU5F1*; NM_002701), and homeobox protein NANOG (*NANOG*; NM_024865), were detected in 30 days hiPSC-RPE cells transfected with *ZNF503-AS1* siRNA (Figure 3c). In addition, mRNA expression levels of RPE markers, including retinoid isomerohydrolase (*RPE65*; NM_000329), retinaldehyde-binding protein 1 (*RLBP1*; NM_000326), lecithin retinol acyltransferase (*LRAT*; NM_004744), tyrosine-protein kinase Mer (*MERTK*; NM_006343), bestrophin-1 (*BEST1*; NM_001139443), cytokeratin-18 (*KRT18*; NM_000224), tight junction protein ZO-1 (*TJP1*; NM_003257), and catenin beta-1 (*CTNNB1*; NM_001904), were tested by real-time PCR. In both 30 d.p.d. hiPSC-RPE and ARPE19 cells, ectopic overexpression of *ZNF503-AS1* elevated mRNA expressions of those RPE markers (Figures 3d and f), while its insufficiency inhibited their expressions (Figures 3e and g).

**Figure 4:**
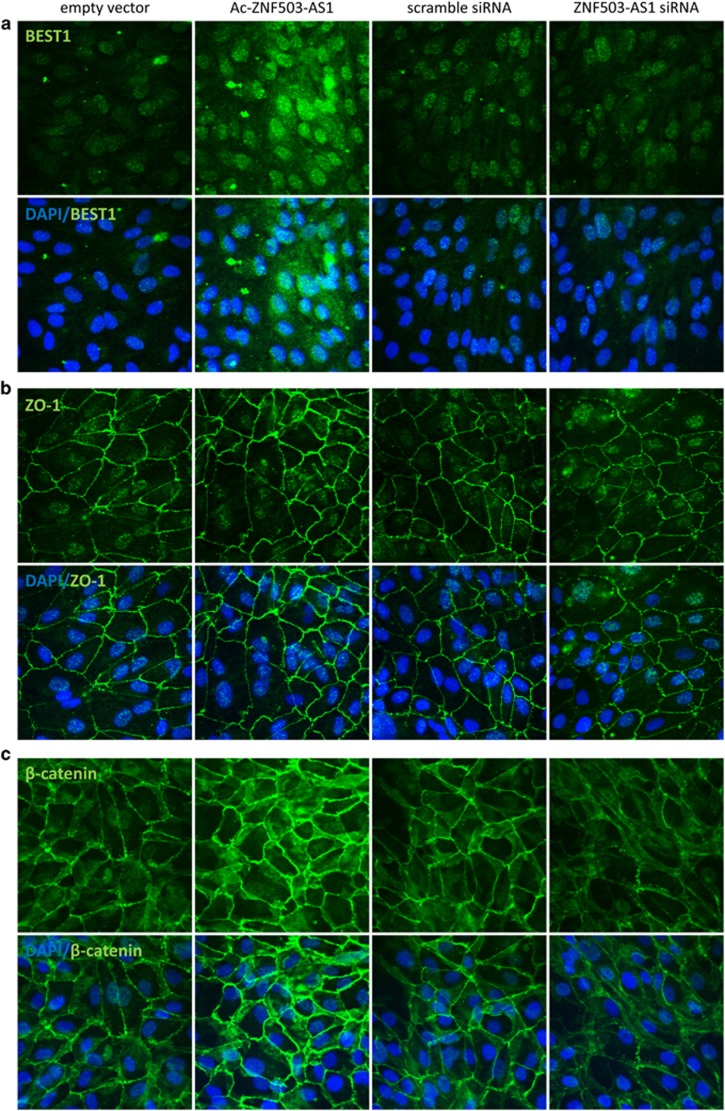
Immunofluorescence of RPE relevant proteins, including bestrophin-1 (**a**), ZO-1 (**b**), and *β*-catenin (**c**). Increased expressions of all three proteins were observed in ARPE19 cells transfected with Ac-*ZNF503-AS1* when compared with cells transfected with empty vector, while decreased protein expressions were detected in cells transfected with *ZNF503-AS1* siRNA compared to cells transfected with scramble siRNA. No obvious mislocalization of proteins was identified in all transfected groups

Immunoblotting further revealed that protein expression levels of Mertk (NP_006334), cytokeratin-18 (NP_000215), ZO-1 (NP_003248), and *β*-catenin (NP_001895) were increased in *ZNF503-AS1*-overexpressed group (Figures 3h and i) while decreased in *ZNF503-AS1*-interfered group (Figures 3h and j). Meanwhile, immunofluorescent staining was used to monitor the intracellular expression and localization of bestrophin-1 (NP_004174), ZO-1, and *β*-catenin. Enhanced expressions of all three proteins were observed in ARPE19 cells overexpressing *ZNF503-AS1* (Figures 4a–c), while decreased protein expressions were detected in cells with *ZNF503-AS1* downregulated. No obvious mislocalization of proteins was identified in all transfected groups. Taken together, our results indicated that *ZNF503-AS1* might contribute to the differentiation of RPE cells.

### *ZNF503-AS1* suppresses RPE proliferation and migration

**Figure 5:**
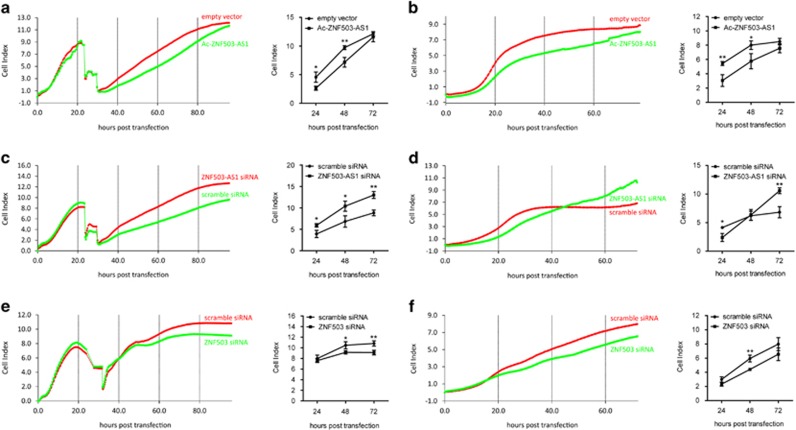
Rates of cell proliferation and migration in different transfected groups. (**a** and **b**) Both proliferation (**a**) and migration (**b**) were inhibited in ARPE19 cells transfected with Ac-*ZNF503-AS1* when compared with cells transfected with empty vector. (**c** and **d**) Rates of proliferation (**c**) and migration (**d**) were increased in ARPE19 cells transfected with *ZNF503-AS1* siRNA compared to cells transfected with scramble siRNA. (**e** and **f**) In cells transfected with *ZNF503* siRNA, rates of both proliferation (**e**) and migration (**f**) were decreased when compared with cells transfected with scramble siRNA

Reportedly, cell proliferation and migration can follow the dedifferentiation of postmitotic tissues, including RPE.^3, 28, 29^ To better reveal the role of *ZNF503-AS1* in RPE function, we tried to determine its effects on RPE proliferation and migration in ARPE19 cells. Rates of cell proliferation and migration were automatically monitored till 72 h post transfection. On the basis of our findings, both proliferation and migration were consistently inhibited in cells transfected with Ac-*ZNF503-AS1* plasmid at all time points post transfection (Figures 5a and b). However, rates of both proliferation and migration elevated in *ZNF503-AS1* siRNA-transfected group (Figures 5c and d). Thus, our data indicated an inhibitory role of *ZNF503-AS1* in RPE proliferation and migration.

### *ZNF503-AS1* is regulated by nuclear factor-*κ*B

**Figure 6:**
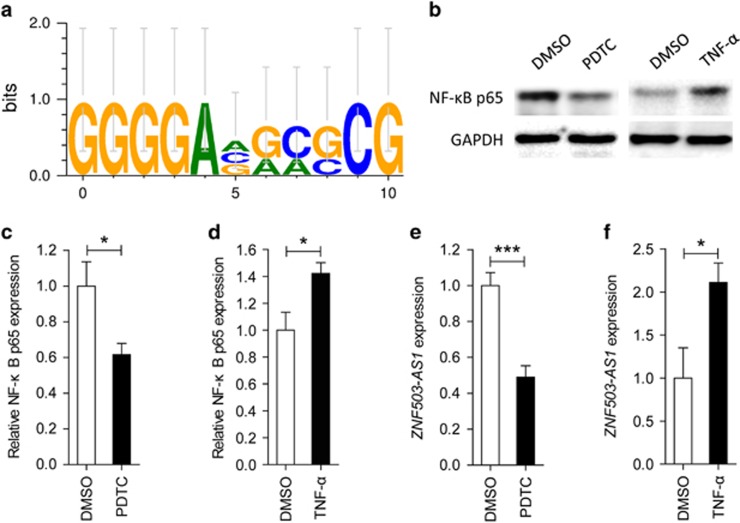
*ZNF503-AS1* is regulated by NF-*κ*B. (**a**) PROMO program predicts NF-*κ*B as a putative transcript factor for *ZNF503-AS1* with three potential TFBS in its promoter region. (**b**–**d**) Protein expression of NF-*κ*B p65, the activated form of NF-*κ*B, was increased in ARPE19 cells treated with PDTC (**b** and **c**) and was decreased in cells treated with TNF-*α* (**b** and **d**) when compared to cells treated with DMSO. (**e** and **f**) mRNA expression of *ZNF503-AS1* was upregulated in ARPE19 cells treated with PDTC (**e**) and was downregulated in cells treated TNF-*α* (**f**) when compared to cells treated with DMSO

We applied PROMO online program to predict transcription factor binding sites (TFBS) in the promoter region of *ZNF503-AS1*. Nuclear factor-*κ*B (NF-*κ*B) was revealed as its putative transcript factor with three potential TFBS in its promoter region (Figure 6a). We then used ammonium pyrrolidinedithiocarbamate (PDTC) to inhibit the activation of NF-*κ*B, and tumor necrosis factor-*α* (TNF-*α*) to stimulate its activation in ARPE19 cells. Expression of NF-*κ*B p65 (activated form of NF-*κ*B) was upregulated in cells treated with PDTC (Figures 6c and d), and was downregulated in cells treated with TNF-*α* (Figures 6b and d). To better understand the regulatory role of NF-*κ*B, we next tested *ZNF503-AS1* expression in different treated groups. Our data supported that expression of *ZNF503-AS1* was reduced in the PDTC-treated group (Figure 6e) and was elevated in the TNF-*α*-treated group (Figure 6f).

### *ZNF503-AS1* regulates *ZNF503* expression

**Figure 7:**
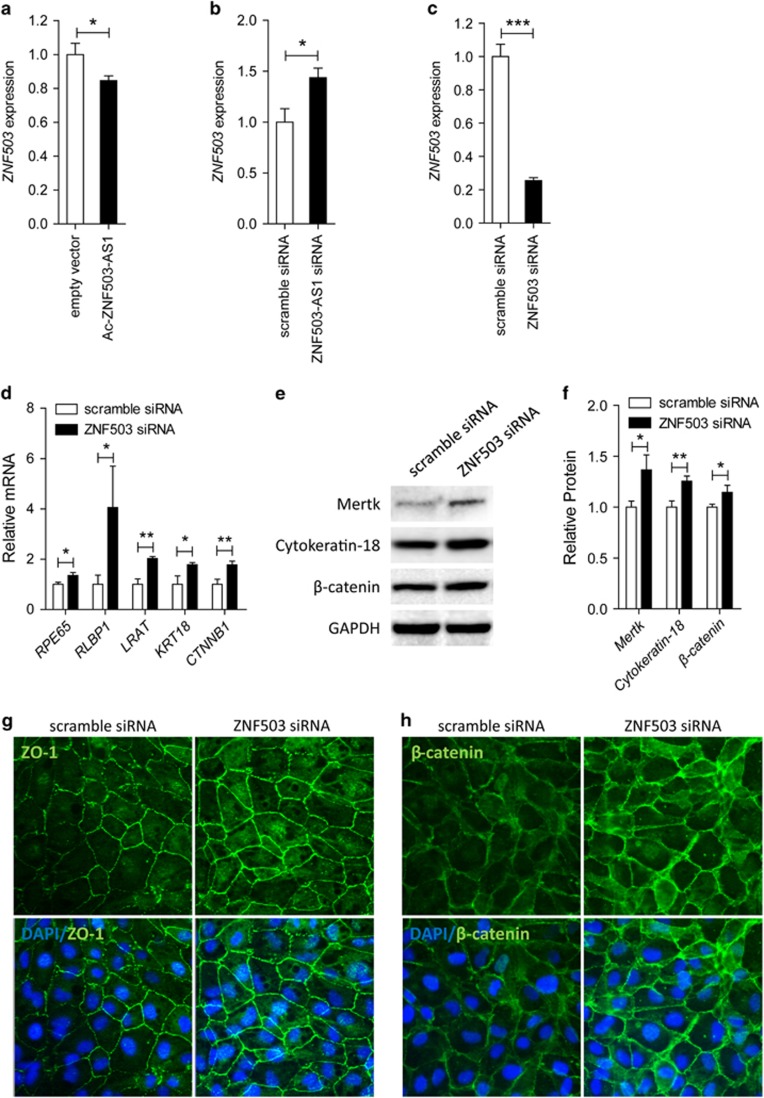
*ZNF503* promotes RPE differentiation, and inhibits cell proliferation and migration. (**a** and **b**) *ZNF503* expression was elevated in ARPE19 cells transfected with Ac-*ZNF503-AS1* when compared to cells transfected with empty vector (**a**), and was reduced in cells transfected with *ZNF503-AS1* siRNA when compared with cells transfected with scramble siRNA (**b**). (**c**) *ZNF503* expression was remarkably decreased in ARPE19 cells transfected with *ZNF503* siRNA. (**d**) In ARPE19 cells transfected with *ZNF503* siRNA, mRNA expressions of RPE relevant markers, including *RPE65*, *RLBP1*, *LRAT*, *KRT18*, and *CTNNB1*, were upregulated. (**e** and **f**) Immunoblotting indicated that protein expressions of Mertk, cytokeratin-18, and *β*-catenin were increased in ARPE19 cells transfected with *ZNF503* siRNA compared to cells transfected with scramble siRNA. (**g** and **h**) Immunofluorescent staining suggested that ZO-1 (**g**) and *β*-catenin (**h**) expressions were elevated in ARPE19 cells transfected with *ZNF503* siRNA when compared to cells transfected with scramble siRNA. No obvious mislocalization of proteins was identified in all transfected groups

*ZNF503-AS1* is transcribed from the antisense strand of the *ZNF503* gene locus. We then assessed whether *ZNF503-AS1* could affect the expression of *ZNF503*. We observed that *ZNF503* mRNA was decreased in cells overexpressing *ZNF503-AS1* (Figure 7a), and was increased in cells with *ZNF503-AS1* knocked down (Figure 7b). Our data suggested a potential regulatory role of *ZNF503-AS1* in *ZNF503* expression.

### *ZNF503* inhibits RPE differentiation, and promotes RPE proliferation and migration

As *ZNF503-AS1* regulates *ZNF503* expression, we next detected whether *ZNF503* insufficiency would improve the regular function of RPE. Endogenous expression of *ZNF503* was remarkably decreased in cells transfected with *ZNF503* siRNA (Figure 7c). Three pairs of siRNA oligos targeting different regions of *ZNF503* were initially designed and tested. We only selected the siRNA showing best efficiency and stability for further investigations (Supplementary Figure S2B). In ARPE19 cells transfected with *ZNF503* siRNA, mRNA expressions of RPE differentiation relevant markers, including *RPE65*, *RLBP1*, *LRAT*, *KRT18*, and *CTNNB1*, were elevated (Figure 7d). Immunoblotting further indicated that *ZNF503* insufficiency in ARPE19 cells would increase the protein expressions of Mertk, cytokeratin-18, and *β*-catenin (Figures 7e and f). Enhanced expressions of ZO-1 and *β*-catenin were also revealed in cells transfected with *ZNF503* siRNA by immunofluorescence (Figures 7g and h). No obvious protein mislocalization was found. Thus, our data indicated an inhibitory role of *ZNF503* in RPE differentiation.

Impacts of *ZNF503* on cell proliferation and migration were also monitored. Our data demonstrated that rates of both proliferation and migration elevated in cells transfected with *ZNF503* siRNA (Figures 5e and f), suggesting that *ZNF503* could promote RPE proliferation and migration. Taken together, our findings indicated that *ZNF503-AS1* promoted RPE differentiation and inhibited its proliferation and migration by interfering with *ZNF503* expression.

## Discussion

RPE dedifferentiation has been typified as a crucial contributing factor to the pathology of atrophic AMD.^1, 3^ Therefore, blocking RPE dedifferentiation is a promising strategy in the treatment of dry AMD. Our group has previously demonstrated that AKT2/mTOR pathway and a group of miRNAs are potentially involved in RPE differentiation.^1, 3^ However, the molecular mechanism underlying RPE differentiation is not fully understood. LncRNAs are important regulators in gene expression. Roles of lncRNA dysregulation in ocular diseases have been well established.^11^ However, its role in RPE differentiation or pathology of atrophic AMD has never been reported. In the present study, we used microarray to obtain lncRNA expression profiles in hiPSC and hiPSC-RPE at different stages. A total of 217 differentially expressed lncRNAs along with the differentiation were initially sorted out. Among all identified lncRNAs, 13 presented a consistent fold change of over 2, including 8 upregulated and 5 downregulated lncRNAs.

*ZNF503-AS1* is an intergenic lncRNA with unrecognized biological interplay. Our data suggest that *ZNF503-AS1* is expressed in the cytoplasm of RPE cells. We have demonstrated that *ZNF503-AS1* is consistently upregulated along with RPE differentiation, and is downregulated in RPE-choroid of atrophic AMD patients and a senior donor with RPE dysfunction. *In vitro* data have also indicated the role of *ZNF503-AS1* in promoting RPE differentiation, and inhibiting RPE proliferation and migration. *ZNF503-AS1* is transcribed from the antisense strand of the *ZNF503* gene locus. *ZNF503* has been recognized as a transcriptional repressor that promotes mammary epithelial cell proliferation and migration, while its role in maintaining RPE function has not been fully elucidated.^30, 31^ RPE cell is a type of mammary epithelial cell. Similar to previous findings, our data indicated that *ZNF503* could inhibit RPE differentiation, and promote its proliferation and migration. We have also revealed that *ZNF503-AS1* expression is reversely correlated with the expression of *ZNF503*. Thus, our findings suggest that *ZNF503-AS1* potentially promotes RPE differentiation by downregulation of *ZNF503* expression. However, how *ZNF503* dysregulation affect RPE function is still poorly understood. Therefore, more investigations are warranted. Thus, our results indicate that *ZNF503-AS1* dysregulation is involved in RPE dedifferentiation and pathology of atrophic AMD, implying its application as a biomarker and therapeutic target for atrophic AMD.

The NF-*κ*B pathway is involved in many cellular processes, including immunity and inflammation. Roles of NF-*κ*B in inflammatory signaling and AMD pathogenesis have been well established.^32, 33, 34^ Activation of the NF-*κ*B pathway has been previously suggested to stimulate pigmentation of cultured primary hRPE cells.^35^ In this study, NF-*κ*B is recognized as a potential upstream transcript factor for *ZNF503-AS1*, which may participate in promoting RPE differentiation by regulating the expression of *ZNF503-AS1*.

Taken together, our study identifies a group of RPE differentiation relevant lncRNAs. We also reveal a potential role of *ZNF503-AS1* in AMD pathogenesis. *ZNF503-AS1* may become a biomarker and a potential therapeutic target for atrophic AMD. However, more investigations are still needed to better illustrate the roles of lncRNAs in RPE differentiation and AMD pathology.

## Materials and methods

### Samples

Postmortem specimens of a 30-year-old female donor and a 70-year-old male donor were provided by Lions New South Wales Eye Bank through Save Sight Institute, the University of Sydney, Australia.^3^ All procedures followed standard procedures of eye donation for research and were approved by the institutional ethical committees conformed to Declaration of Helsinki. Written informed consents were obtained from all donors before their donations.

### Culture and differentiation of hiPSC-RPE

HiPSC (IMR90-57) were cultured on mouse embryonic fibroblasts (SiDan-Sai Biotechnology Co., Ltd, Shanghai, China) in six-well tissue culture plates as detailed elsewhere.^3^ The hiPSCs were then differentiated into RPE cells according to the SFEB/CS method using low-molecular-weight compounds CKI-7 (5 *μ*M) and SB-431542 (5 *μ*M).^36^

### Microarray profiling and computational analysis

Total RNA was isolated from hiPSC-RPE cells at different stages using TRIzol reagent (Invitrogen, Carlsbad, CA, USA).^4, 37^ Qualities and concentrations of RNA samples were determined using Nano-Drop ND-1000 spectrophotometer (Nano-Drop Technologies, Wilmington, DE, USA). Agilent Sureprint G3 Human GE 8 × 60 K Microarray (Agilent Co., Palo Alto, CA, USA) covering 27958 Entrez Gene RNAs and 7419 human lncRNAs was used to generate the lncRNA expression profiles from the following four groups in duet: hiPSC; and hiPSC-RPE at 30, 60, and 90 d.p.d. An Aglient G3 scanner was then used to determine the immunofluorescence intensities of the arrays. Data were further analyzed using feature extraction software. Multiples of differentially expressed genes were calculated. miRNA expression profiles of the four groups were generated as described before.^3^ In addition, microarray data (GSE29801) and sample information (GSM738433–GSM738607) of 175 independent RPE-choroid samples were downloaded from Gene Expression Omnibus data sets and analyzed as indicated previously.^3, 27^ Among the 175 samples, 88 were obtained from normal individuals, including 50 macular and 38 extramacular samples, and 87 were from AMD patients, including 41 macular and 46 extramacular samples.

### Reverse transcription-PCR and real-time PCR

Reverse transcription-PCR (RT-PCR) and real-time PCR were performed using a previously defined protocol.^4, 37^ One microgram of the extracted total RNA was used for cDNA synthesis using PrimeScript RT Kit (Takara, Otsu, Japan). Real-time PCR was conducted using FastStart Universal SYBR Green Master (ROX; Roche, Basel, Switzerland) with StepOne Plus Real-time PCR System (Applied Biosystems, Darmstadt, Germany). Information of primers was provided in Supplementary Table S2.

### Bioinformatics analysis

To enhance data credibility, only lncRNAs whose sequences had been recorded in ENCODE were included in the present study.^38^ Genomic location and conservation of lncRNA, *ZNF503-AS1* (ENST00000416398), was obtained from UCSC genome browser (http://genome.ucsc.edu/index.html).^39^ We used PROMO (http://alggen.lsi.upc.es/cgi-bin/promo_v3/promo/promoinit.cgi?dirDB=TF_8.3) to identify putative TFBS in the promoter region of the lncRNA.^40, 41^ Results were demonstrated using WebLogo 3 (http://weblogo.threeplusone.com/).^42, 43^

### Fluorescent *in situ* hybridization

*ZNF503-AS1*, U6, and 18 S FISH probes were synthesized by RiboBio (Guangzhou, China). FISH was conducted using the FISH kit per the manufacturer’s protocol (RiboBio). Briefly, ARPE19 cells were collected, fixed with 4% paraformaldehyde, permeabilized in 0.5% Triton X-100 on ice, and then treated with pre-hybridization buffer. We next hybridized cells with Cy3-labeled RNA of *ZNF503-AS1* probe mix in a moist chamber. Cells were then stained with 4′,6-diamidino-2-phenylindole (DAPI). Images were collected using a confocal microscope (LSM 510; Carl Zeiss, Jena, Germany).

### Reagents and cell transfection

*ZNF503-AS1* and *ZNF503* siRNAs were purchased from RiboBio Co. Ltd. Open reading frame sequence of *ZNF503-AS1* was synthesized, amplified, and inserted into the expression vector pcDNA3.1 (Sigma, St. Louis, MO, USA) using *Xho*I and *Kpn*I restriction sites to generate the Ac-ZNF503-AS1 plasmids. Sequences of the constructed plasmids were confirmed using Sanger sequencing.

For transfection assay, cells were seeded into six-well plates and transfected with 100 pmol siRNA or 4 *μ*g expression vector using Lipofectamine 2000 transfection reagent (Invitrogen) per the manufacturer’s protocol. Cells were collected at 48 h post transfection for RNA isolation, and at 72 h post transfection for protein extraction and immunofluorescent staining. PDTC (Beyotime, Shanghai, China; concentration: 100 *μ*M)^44^ and TNF-*α* (Sigma; concentration: 100 ng/*μ*l) were used for cell stimulation. Cells were collected at 48 h after treatment.

### Immunoblotting

Immunoblotting was performed per previously described protocols.^3, 45^ Briefly, cells were collected at 72 h post transfection in ice-cold RIPA buffer (Beyotime) containing protease inhibitors cocktail (Roche) for protein extraction. Extracted proteins were separated by 10% SDS-polyacrylamide gel electrophoresis and transferred to a polyvinylidene fluoride membrane (Millipore, Billerica, MA, USA). Membranes were then blocked, incubated with primary antibodies at 4 °C overnight (Supplementary Table S3), washed, and probed with corresponding horseradish peroxidase-conjugated secondary antibodies (1:10 000 diluted in 1 × PBS; ICL Inc., Newberg, OR, USA) for 1 h at room temperature. Autoradiography with the ECL-Western blotting system (BioRad, Hercules, CA, USA) was then applied to develop the blots per manufacturers’ protocols. ImageJ software (http://rsb.info.nih.gov/ij/index.html) was used to determine and quantify protein expressions.

### Immunofluorescent staining

Immunofluorescence was performed per previously described protocols.^3, 45^ Cells were collected, fixed with 4% paraformaldehyde, permeabilized with 0.5% Triton X-100, blocked in bovine serum albumin, and incubated with primary antibodies (Supplementary Table S3) at 4 °C overnight. Cells were then washed and incubated with corresponding fluorescence-conjugated secondary antibodies (1:1000 diluted in 1 × PBS; Invitrogen) for 1 h at room temperature. Cell nuclei were counterstained by DAPI (Sigma). Images were collected using a confocal microscope (LSM 510).

### Monitoring cell proliferation and migration

Rates of cell proliferation and migration were detected in real-time using xCELLigence system E-Plate (Roche) according to the manufacturer’s protocol. To monitor cell proliferative rates, ~5000 cells were seeded in each well of the E-Plate. Transfection was performed at 24 h post plantation. For migration assay, 40 000 post-transfection cells were planted into each well. Cells were culture with fresh DME/F12 medium. Impedance value for each well was automatically determined by the xCELLigence system and expressed as a CI value. Rates of cell proliferation and migration were determined by calculating the slope of the line between two given time points.

### Statistics

We used GraphPad Prism (version 4.0; GraphPad Software, San Diego, CA, USA) for statistical analysis. Student’s *t*-test was used for comparisons between groups. All presented data were based on biological triplicates. Data were shown as mean±S.D., and *P*-value <0.05 was considered as statistically significant.

## Publisher’s Note

Springer Nature remains neutral with regard to jurisdictional claims in published maps and institutional affiliations.

## Figures and Tables

**Table 1:** Differentially expressed lncRNAs along with the differentiation with consistent fold change of over 2

**lncRNAs**	**Gene ID**	**Expression change**	**Fold changes compared to undifferentiated hiPSC**		
			**30 d.p.d.**	**60 d.p.d.**	**90 d.p.d.**
*RP11-367G18.1*	ENSG00000230943	Upregulated	12.33913	97.291084	240.70334
*CTD-2319I12.1*	ENSG00000261040	Upregulated	6.7098174	78.71717	175.4076
*RP3-395M20.8*	ENSG00000238164	Upregulated	6.921395	68.56575	139.77567
*RP11-1020M18.10*	ENSG00000257500	Upregulated	5.608035	27.175821	97.06175
*H19*	ENSG00000130600	Upregulated	8.185132	27.15535	55.142082
*RP11-195B3.1*	ENSG00000227338	Upregulated	4.79346	19.776524	40.09696
*RP11-554I8.2*	ENSG00000223784	Upregulated	4.205783	14.769911	32.634445
*ZNF503-AS1*	ENSG00000226051	Upregulated	4.1409206	11.061297	29.609468
*ESRG*	ENSG00000265992	Downregulated	0.04946436	0.0014118	0.000272751
*LINC00617*	ENSG00000250366	Downregulated	0.023696999	0.007646307	0.003706984
*RP11-469A15.2*	ENSG00000230623	Downregulated	0.098930394	0.033249393	0.010706429
*LINC01162*	ENSG00000232790	Downregulated	0.484361587	0.119291044	0.024939111
*LINC00173*	ENSG00000196668	Downregulated	0.323728262	0.138572432	0.04917562
