# Supplementary figures and images for: LncRNA ZNF503-AS1 promotes RPE differentiation by downregulating ZNF503 expression
Source: Cell Death Dis. 2017 Sep 7;8(9):e3046–. doi: 10.1038/cddis.2017.382 (PMC5636965; doi:10.1038/cddis.2017.382)

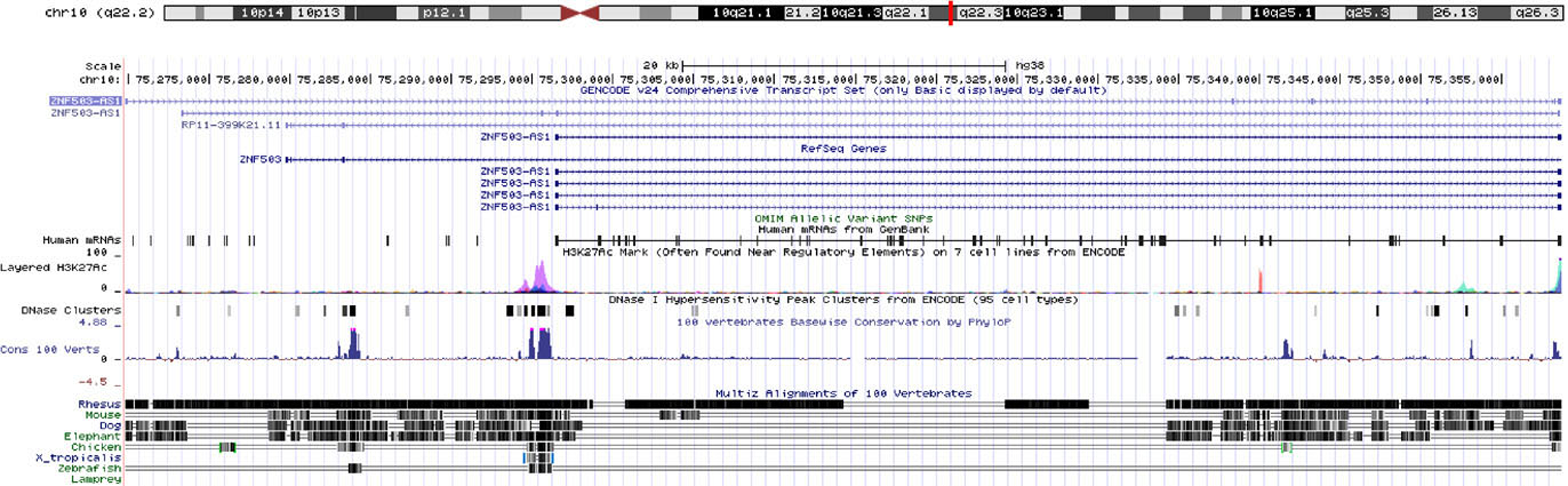

Supplement: Supplementary Figure S1 [file cddis2017382x1.tif]

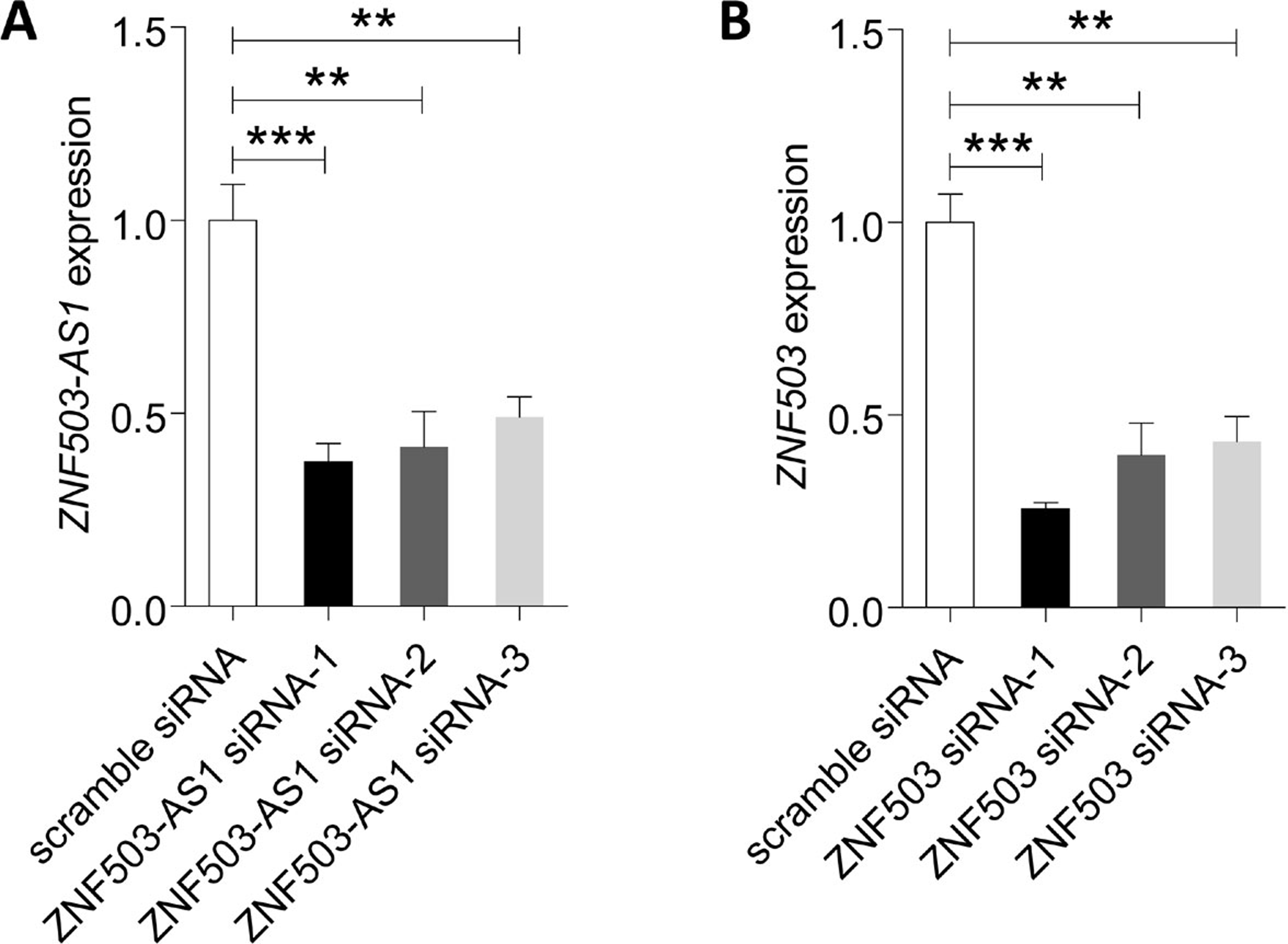

Supplement: Supplementary Figure S2 [file cddis2017382x2.tif]
