# Supplementary material for: LncRNA ZNF503-AS1 promotes RPE differentiation by downregulating ZNF503 expression
Source: Cell Death Dis. 2017 Sep 7;8(9):e3046–. doi: 10.1038/cddis.2017.382 (PMC5636965; doi:10.1038/cddis.2017.382)
Supplement: Supplementary Materials [file cddis2017382x3.docx]

**Legends to Supplementary Figures**

**Figure S1.** Chromosome location of *ZNF503-AS1* in human genome.

**Figure S2.** Knockdown efficiencies of three pairs of siRNAs oligos targeting different regions of the *ZNF503-AS1* gene (**A**) and the *ZNF503* gene (**B**).

| **Supplementary Table S1. A collection of all differentially expressed lncRNAs along with the differentiation.** | | | | |
| --- | --- | --- | --- | --- |
| **LncRNAs** | **Expression change** | **Mean relative expression of hiPSC-RPE to hiPSC** | | |
|  |  | **30 dpd** | **60 dpd** | **90 dpd** |
| *PART1* | Up-regulated | 316.82928 | 514.31854 | 692.00287 |
| *RP11-486L19.2* | Up-regulated | 29.978416 | 222.2068 | 335.9337 |
| *RP11-151D14.1* | Up-regulated | 2.1866498 | 282.4854 | 290.01297 |
| *RP11-95M15.1* | Up-regulated | 95.51913 | 151.59578 | 261.40463 |
| *RP11-367G18.1* | Up-regulated | 12.33913 | 97.291084 | 240.70334 |
| *LINC00478* | Up-regulated | 30.2896 | 168.1393 | 234.66847 |
| *RP11-527N22.1* | Up-regulated | 6.697713 | 88.90941 | 181.07965 |
| *CTD-2319I12.1* | Up-regulated | 6.7098174 | 78.71717 | 175.4076 |
| *RP3-395M20.8* | Up-regulated | 6.921395 | 68.56575 | 139.77567 |
| *AP000473.5* | Up-regulated | 20.940847 | 91.90864 | 113.588234 |
| *RP11-1020M18.10* | Up-regulated | 5.608035 | 27.175821 | 97.06175 |
| *RP11-400K9.4* | Up-regulated | 10.014255 | 47.65581 | 85.08897 |
| *LINC00908* | Up-regulated | 4.7252693 | 68.41397 | 72.375244 |
| *RP11-544L8__B.4* | Up-regulated | 12.373166 | 38.928913 | 70.726166 |
| *ZNF295-AS1* | Up-regulated | 9.068667 | 38.17337 | 60.35985 |
| *H19* | Up-regulated | 8.185132 | 27.15535 | 55.142082 |
| *RP11-123B3.2* | Up-regulated | 18.618286 | 31.078741 | 49.384163 |
| *HOXB-AS3* | Up-regulated | 8.419304 | 47.825294 | 48.87888 |
| *LINC01158* | Up-regulated | 8.17595 | 37.790474 | 48.40495 |
| *RP11-372M18.2* | Up-regulated | 5.0684 | 16.05424 | 45.026054 |
| *RP11-195B3.1* | Up-regulated | 4.79346 | 19.776524 | 40.09696 |
| *CTA-150C2.13* | Up-regulated | 12.357811 | 36.643116 | 39.2354 |
| *PROX1-AS1* | Up-regulated | 12.679336 | 29.09205 | 38.82854 |
| *KRT42P* | Up-regulated | 15.684065 | 27.392511 | 38.534996 |
| *AC019117.1* | Up-regulated | 7.0937715 | 31.281773 | 36.01937 |
| *MEIS1-AS3* | Up-regulated | 15.134532 | 26.724258 | 35.54771 |
| *CTC-529L17.1* | Up-regulated | 5.2329283 | 24.31634 | 33.582947 |
| *RP11-554I8.2* | Up-regulated | 4.205783 | 14.769911 | 32.634445 |
| *ZNF503-AS1* | Up-regulated | 4.1409206 | 11.061297 | 29.609468 |
| *LINC00511* | Up-regulated | 10.291572 | 15.745048 | 29.518145 |
| *RP11-521D12.1* | Up-regulated | 8.9156685 | 21.984133 | 23.015125 |
| *RP11-669N7.2* | Up-regulated | 5.1446514 | 17.648647 | 22.752764 |
| *DNAJC27-AS1* | Up-regulated | 6.6903563 | 19.8516 | 22.298779 |
| *RP11-1C1.7* | Up-regulated | 3.6992805 | 6.334754 | 21.855963 |
| *LINC00323* | Up-regulated | 3.1307995 | 18.999292 | 21.207445 |
| *AC008278.2* | Up-regulated | 10.173906 | 15.685569 | 19.8311 |
| *LINC00354* | Up-regulated | 4.822219 | 17.529303 | 19.145546 |
| *RP5-1021I20.1* | Up-regulated | 10.847839 | 17.959972 | 18.958075 |
| *LINC01116* | Up-regulated | 5.174996 | 13.108533 | 18.630795 |
| *RP11-503G7.1* | Up-regulated | 5.94477 | 17.846088 | 18.508137 |
| *RP11-54A9.1* | Up-regulated | 4.444495 | 9.023744 | 17.695965 |
| *LINC01094* | Up-regulated | 3.9005508 | 5.2393856 | 17.206602 |
| *HOTAIRM1* | Up-regulated | 15.240413 | 15.891515 | 17.165504 |
| *AC011298.2* | Up-regulated | 8.610012 | 16.608273 | 17.016928 |
| *RP11-159K7.2* | Up-regulated | 8.691094 | 10.921713 | 16.653362 |
| *AC004069.2* | Up-regulated | 5.710119 | 9.660374 | 15.919135 |
| *RP3-395M20.9* | Up-regulated | 2.0296671 | 13.589864 | 15.48137 |
| *AC003075.4* | Up-regulated | 11.978225 | 12.26988 | 15.240866 |
| *AC007556.3* | Up-regulated | 6.8472767 | 9.209429 | 15.14843 |
| *RP11-187C18.3* | Up-regulated | 6.0376673 | 13.752068 | 15.120253 |
| *RP11-345L23.1* | Up-regulated | 4.7156396 | 5.1266437 | 14.91289 |
| *lncRNA* | Up-regulated | 3.0527337 | 10.676343 | 14.379034 |
| *TTTY2* | Up-regulated | 8.022979 | 11.019658 | 13.729991 |
| *LINC00278* | Up-regulated | 10.754919 | 11.002731 | 13.686983 |
| *RP11-344B5.2* | Up-regulated | 2.4574785 | 7.7689047 | 12.728244 |
| *AC006262.6* | Up-regulated | 2.4431357 | 6.2513094 | 12.184157 |
| *AC007879.7* | Up-regulated | 4.368788 | 6.5342026 | 12.044081 |
| *LINC00284* | Up-regulated | 2.750181 | 11.635748 | 12.017967 |
| *LINC00683* | Up-regulated | 7.1495833 | 10.442005 | 11.907465 |
| *RP5-1185K9.1* | Up-regulated | 4.958172 | 6.4147525 | 11.056838 |
| *IL10RB-AS1* | Up-regulated | 3.2189713 | 3.4617157 | 10.808564 |
| *RP11-150O12.1* | Up-regulated | 3.2783465 | 5.574656 | 10.685927 |
| *MIR31HG* | Up-regulated | 8.829125 | 10.342257 | 10.661036 |
| *RP5-942I16.1* | Up-regulated | 3.1004186 | 6.1009126 | 10.649294 |
| *LINC00963* | Up-regulated | 7.0815268 | 9.793755 | 10.624687 |
| *AC018647.3* | Up-regulated | 5.1454577 | 6.8960347 | 10.26938 |
| *RP11-119J18.1* | Up-regulated | 2.5251992 | 10.085151 | 10.262197 |
| *MAST4-AS1* | Up-regulated | 3.3252017 | 4.858066 | 10.016254 |
| *RP11-356J5.12* | Up-regulated | 5.156002 | 9.866989 | 9.935986 |
| *RP11-646E18.2* | Up-regulated | 2.4493687 | 3.0562813 | 9.799074 |
| *RP4-666F24.3* | Up-regulated | 2.49042 | 2.5630558 | 9.388291 |
| *DPH6-AS1* | Up-regulated | 3.6990886 | 9.087387 | 9.264725 |
| *RP11-379F4.4* | Up-regulated | 2.0497677 | 6.8606696 | 9.169358 |
| *AC007743.1* | Up-regulated | 3.6011992 | 7.5770793 | 9.1519575 |
| *CTD-2540L5.6* | Up-regulated | 2.9226305 | 6.762445 | 9.107531 |
| *RP11-288L9.1* | Up-regulated | 3.9837096 | 6.152566 | 8.918876 |
| *RP11-114H23.1* | Up-regulated | 3.4491744 | 7.710967 | 8.469974 |
| *RP1-153P14.8* | Up-regulated | 2.9476945 | 5.3706083 | 8.044678 |
| *AC003090.1* | Up-regulated | 3.9954903 | 4.5827837 | 8.01079 |
| *Z83851.4* | Up-regulated | 4.8078623 | 5.730434 | 7.7380886 |
| *RP11-150O12.6* | Up-regulated | 3.8800075 | 3.9688785 | 7.2678967 |
| *RP1-124C6.1* | Up-regulated | 2.3650613 | 2.497093 | 7.063751 |
| *RP1-43E13.2* | Up-regulated | 3.3314798 | 6.031299 | 6.8103833 |
| *AC046143.3* | Up-regulated | 2.6840885 | 4.546895 | 6.421507 |
| *RP11-770J1.3* | Up-regulated | 4.55787 | 5.8021045 | 6.14264 |
| *LINC01091* | Up-regulated | 2.087907 | 3.892952 | 5.5337663 |
| *CTC-228N24.3* | Up-regulated | 4.3690867 | 5.1562204 | 5.3231335 |
| *RP11-61J19.3* | Up-regulated | 2.568079 | 3.3721814 | 5.133261 |
| *RP11-359B12.2* | Up-regulated | 3.3223894 | 4.5145493 | 5.0564494 |
| *LINC00324* | Up-regulated | 3.2135265 | 4.4053974 | 4.8537536 |
| *RP11-573G6.6* | Up-regulated | 2.9480462 | 3.4663675 | 4.8407364 |
| *RP11-838N2.4* | Up-regulated | 2.1562297 | 4.3538036 | 4.77108 |
| *RP11-501O2.5* | Up-regulated | 2.2388382 | 4.5836167 | 4.7622085 |
| *ZBED5-AS1* | Up-regulated | 2.9280643 | 4.0054 | 4.4972425 |
| *LINC01140* | Up-regulated | 2.9695637 | 3.3588352 | 4.3747497 |
| *RP11-503N18.1* | Up-regulated | 2.2634585 | 2.8934586 | 4.343699 |
| *RP11-206L10.10* | Up-regulated | 2.477496 | 2.9745667 | 4.1169376 |
| *RP11-244K5.8* | Up-regulated | 3.7460427 | 4.053581 | 4.0875816 |
| *AC017076.5* | Up-regulated | 2.7305744 | 3.6870728 | 4.0447283 |
| *AC079586.1* | Up-regulated | 2.9185522 | 3.433453 | 4.0270762 |
| *RP11-789C17.3* | Up-regulated | 2.1690848 | 3.2588456 | 3.8907754 |
| *RP11-67L2.2* | Up-regulated | 2.2844672 | 3.068049 | 3.8467681 |
| *ARHGEF26-AS1* | Up-regulated | 2.066944 | 3.2905092 | 3.761539 |
| *AC108488.4* | Up-regulated | 3.4391193 | 3.61063 | 3.7286258 |
| *RP11-513G11.4* | Up-regulated | 2.1279233 | 2.573757 | 3.6863987 |
| *BOK-AS1* | Up-regulated | 3.0896282 | 3.41343 | 3.6827745 |
| *LINC00969* | Up-regulated | 2.595136 | 3.5571089 | 3.6318712 |
| *CTD-2201E18.3* | Up-regulated | 2.9834628 | 3.156211 | 3.63048 |
| *LINC00230A* | Up-regulated | 2.5029564 | 2.9096458 | 3.4674962 |
| *LINC00035-001* | Up-regulated | 2.002513 | 2.006199 | 3.2639732 |
| *CCDC13-AS1* | Up-regulated | 2.4898257 | 2.9096081 | 3.1765914 |
| *GS1-251I9.4* | Up-regulated | 2.0309782 | 2.9124763 | 3.157369 |
| *RP11-297K8.2* | Up-regulated | 2.3856385 | 2.583562 | 3.1546464 |
| *RP11-494M8.4* | Up-regulated | 2.1310778 | 2.7107527 | 3.0793877 |
| *RP5-857K21.4* | Up-regulated | 2.3842835 | 2.8742085 | 3.0064886 |
| *NOVA1-AS1* | Up-regulated | 2.1501052 | 2.2481866 | 2.5393732 |
| *ESRG* | Down-regulated | 0.04946436 | 0.0014118 | 0.000272751 |
| *LINC00678* | Down-regulated | 0.002252963 | 0.001087503 | 0.000799961 |
| *LINC00545* | Down-regulated | 0.02108775 | 0.003304645 | 0.001708818 |
| *RP1-241P17.1* | Down-regulated | 0.02139219 | 0.002978414 | 0.002257935 |
| *LINC00698* | Down-regulated | 0.005239449 | 0.003588679 | 0.002901829 |
| *LINC00617* | Down-regulated | 0.023696999 | 0.007646307 | 0.003706984 |
| *LINC00428* | Down-regulated | 0.008613662 | 0.005716342 | 0.004406107 |
| *CTD-2354A18.1* | Down-regulated | 0.041905475 | 0.007395738 | 0.005679081 |
| *RP11-100E13.1* | Down-regulated | 0.038959914 | 0.009690022 | 0.008052437 |
| *RP11-469A15.2* | Down-regulated | 0.090227602 | 0.013925274 | 0.008403894 |
| *RP11-849I19.1* | Down-regulated | 0.067616411 | 0.016027493 | 0.009916836 |
| *RP5-964N17.1* | Down-regulated | 0.030017843 | 0.012695134 | 0.011406236 |
| *RP11-429A20.4* | Down-regulated | 0.056860355 | 0.026613241 | 0.014865517 |
| *CTD-2306M5.1* | Down-regulated | 0.35245852 | 0.018828048 | 0.016383263 |
| *LINC01162* | Down-regulated | 0.484361587 | 0.119291044 | 0.024939111 |
| *RP11-893F2.6* | Down-regulated | 0.270732081 | 0.055886497 | 0.028216609 |
| *SNHG14* | Down-regulated | 0.069273915 | 0.055675217 | 0.036240988 |
| *AC113617.1* | Down-regulated | 0.069089325 | 0.047077382 | 0.036452829 |
| *AC005592.1* | Down-regulated | 0.052292099 | 0.041302636 | 0.039767876 |
| *AP000459.4* | Down-regulated | 0.086210925 | 0.05420066 | 0.04124834 |
| *AC005062.2* | Down-regulated | 0.082681363 | 0.077463828 | 0.041386754 |
| *HCG24* | Down-regulated | 0.091818292 | 0.047678266 | 0.042923374 |
| *RP11-98G7.1* | Down-regulated | 0.100224212 | 0.067113283 | 0.043829317 |
| *RP11-799P8.1* | Down-regulated | 0.055209493 | 0.055134986 | 0.045765658 |
| *HPN-AS1* | Down-regulated | 0.185080717 | 0.069248371 | 0.048054737 |
| *RP11-435B5.5* | Down-regulated | 0.100555125 | 0.085499894 | 0.048764108 |
| *LINC00173* | Down-regulated | 0.323728262 | 0.138572432 | 0.04917562 |
| *AC116614.1* | Down-regulated | 0.248017189 | 0.131559577 | 0.050096938 |
| *LINC00633* | Down-regulated | 0.128261948 | 0.077340113 | 0.051987673 |
| *LINC00371* | Down-regulated | 0.087018797 | 0.064450755 | 0.056156496 |
| *LINC00337* | Down-regulated | 0.308995317 | 0.096997858 | 0.060989392 |
| *lincROR* | Down-regulated | 0.094295153 | 0.092144408 | 0.067828427 |
| *AC012668.2* | Down-regulated | 0.158062058 | 0.091891799 | 0.069894863 |
| *AC002115.5* | Down-regulated | 0.173452474 | 0.096304451 | 0.071925738 |
| *XIST* | Down-regulated | 0.148674619 | 0.116484597 | 0.075180226 |
| *RP11-438N16.1* | Down-regulated | 0.230032728 | 0.104282453 | 0.075624631 |
| *PVT1* | Down-regulated | 0.305851253 | 0.112142033 | 0.086584697 |
| *CTC-523E23.6* | Down-regulated | 0.264824568 | 0.253069787 | 0.087545099 |
| *RP11-20J15.3* | Down-regulated | 0.195275047 | 0.143883435 | 0.088267868 |
| *RP11-1M18.1* | Down-regulated | 0.220330688 | 0.091285153 | 0.090779318 |
| *RP11-390F4.6* | Down-regulated | 0.158050516 | 0.157093427 | 0.093556992 |
| *RP11-542F9.1* | Down-regulated | 0.32821576 | 0.271981978 | 0.104554061 |
| *RP11-486O13.4* | Down-regulated | 0.16044707 | 0.130237637 | 0.106061635 |
| *LINC00659* | Down-regulated | 0.146294827 | 0.12156825 | 0.106993227 |
| *AF131215.4* | Down-regulated | 0.471080309 | 0.387187051 | 0.108208116 |
| *AP000688.29* | Down-regulated | 0.19972507 | 0.1128069 | 0.108660196 |
| *PWRN1* | Down-regulated | 0.399109921 | 0.254022704 | 0.109080874 |
| *RP11-89M16.1* | Down-regulated | 0.36004828 | 0.122574894 | 0.109975976 |
| *AC007386.4* | Down-regulated | 0.394245964 | 0.204128311 | 0.121793749 |
| *RP11-453F18__B.1* | Down-regulated | 0.210116774 | 0.182820112 | 0.124734969 |
| *RP11-101E14.2* | Down-regulated | 0.464298854 | 0.165971468 | 0.129242281 |
| *GNAS-AS1* | Down-regulated | 0.332087011 | 0.222761007 | 0.132442928 |
| *AC074363.1* | Down-regulated | 0.316310874 | 0.230419875 | 0.13333904 |
| *RP11-22P4.1* | Down-regulated | 0.260644513 | 0.19161843 | 0.135717578 |
| *ITPK1-AS1* | Down-regulated | 0.341156449 | 0.271254977 | 0.1369432 |
| *AC096670.3* | Down-regulated | 0.309421584 | 0.186823792 | 0.137912096 |
| *FEZF1-AS1* | Down-regulated | 0.300211472 | 0.192984239 | 0.140188377 |
| *RP11-82C23.2* | Down-regulated | 0.254578778 | 0.149197348 | 0.140326056 |
| *RP1-212P9.3* | Down-regulated | 0.320102207 | 0.174100072 | 0.157675823 |
| *AC156455.1* | Down-regulated | 0.355180851 | 0.17791931 | 0.158539484 |
| *AP001628.7* | Down-regulated | 0.430465915 | 0.214536633 | 0.161179083 |
| *RP11-461K13.1* | Down-regulated | 0.224467697 | 0.22223004 | 0.172582576 |
| *SLC16A1-AS1* | Down-regulated | 0.359593745 | 0.309855 | 0.172743626 |
| *LINC00309* | Down-regulated | 0.235137621 | 0.226438046 | 0.173725239 |
| *RP1-40E16.2* | Down-regulated | 0.420201224 | 0.2458347 | 0.174368144 |
| *RP11-547D24.1* | Down-regulated | 0.411484313 | 0.374139031 | 0.176623795 |
| *AC007386.2* | Down-regulated | 0.296072106 | 0.266840305 | 0.179683754 |
| *RP11-1023L17.1* | Down-regulated | 0.331681031 | 0.279141695 | 0.181127985 |
| *FAM201A* | Down-regulated | 0.369061841 | 0.223613696 | 0.1829681 |
| *RP11-340E6.1* | Down-regulated | 0.245125255 | 0.241720102 | 0.185121524 |
| *RP1-20B11.2* | Down-regulated | 0.476639743 | 0.193087793 | 0.186282781 |
| *RP11-775H9.2* | Down-regulated | 0.351446129 | 0.215603683 | 0.190058254 |
| *RP11-69I8.3* | Down-regulated | 0.429132133 | 0.212767497 | 0.19561161 |
| *DGCR5* | Down-regulated | 0.292485723 | 0.240364375 | 0.196308046 |
| *RP11-586D19.1* | Down-regulated | 0.336568542 | 0.28069627 | 0.19698848 |
| *AC010967.2* | Down-regulated | 0.459521551 | 0.254953654 | 0.199650492 |
| *RP11-656A15.1* | Down-regulated | 0.401935722 | 0.308365227 | 0.203836376 |
| *JPX* | Down-regulated | 0.446594988 | 0.311717851 | 0.220912735 |
| *RP11-225N10.1* | Down-regulated | 0.424387874 | 0.316332417 | 0.220915644 |
| *KTN1-AS1* | Down-regulated | 0.303540647 | 0.255195461 | 0.221117641 |
| *GRM7-AS3* | Down-regulated | 0.467179561 | 0.235197216 | 0.22144777 |
| *RP11-1112C15.1* | Down-regulated | 0.339669044 | 0.279694618 | 0.222972475 |
| *DLGAP1-AS2* | Down-regulated | 0.395360758 | 0.330860296 | 0.227315535 |
| *AC104389.16* | Down-regulated | 0.372610644 | 0.348944287 | 0.232497566 |
| *RP5-1074L1.1* | Down-regulated | 0.475522842 | 0.265050677 | 0.236376723 |
| *AP000688.15* | Down-regulated | 0.438236349 | 0.319152708 | 0.240733679 |
| *RP11-554K11.2* | Down-regulated | 0.421290612 | 0.349355462 | 0.256652391 |
| *RP11-305L7.1* | Down-regulated | 0.352613014 | 0.309658141 | 0.25871517 |
| *RP11-85L21.4* | Down-regulated | 0.414738874 | 0.316517417 | 0.261577519 |
| *CASC15* | Down-regulated | 0.499134127 | 0.380011646 | 0.27643449 |
| *RP11-380P13.2* | Down-regulated | 0.452929382 | 0.309689145 | 0.278789754 |
| *RP11-722M1.1* | Down-regulated | 0.454969279 | 0.325340646 | 0.286014364 |
| *CASC14* | Down-regulated | 0.462207468 | 0.375565343 | 0.288391681 |
| *RP11-452K12.4* | Down-regulated | 0.471777247 | 0.304054534 | 0.291234952 |
| *LINC00937* | Down-regulated | 0.484500584 | 0.420268843 | 0.293523392 |
| *CTD-2616J11.3* | Down-regulated | 0.440445881 | 0.412170573 | 0.336051215 |
| *RP11-360K13.1* | Down-regulated | 0.458126504 | 0.440553476 | 0.337533396 |
| *RP11-344B5.3* | Down-regulated | 0.460102997 | 0.34406921 | 0.340273356 |
| *OIP5-AS1* | Down-regulated | 0.379366211 | 0.363123277 | 0.362178995 |
| *RP4-535B20.1* | Down-regulated | 0.481645455 | 0.428342846 | 0.37669948 |
| *TTTY15* | Down-regulated | 0.485737296 | 0.465897839 | 0.444324674 |

| **Supplementary Table S2. Primers used in this study** | | |
| --- | --- | --- |
| **LncRNA/mRNA** | **Forward Primer (5'→3')** | **Reverse Primer (5'→3')** |
| *GAPDH* | CAGCCTCAAGATCATCAGCA | TGTGGTCATGAGTCCTTCCA |
| *RP11-367G18.1* | GGCCCTTGTGAATTGATGA | GGGCCACATAGCAAGATCC |
| *CTD-2319I12.1* | AACCCCGGAGACCACTGT | ACTCCAGACAGTCACATCTTGG |
| *RP3-395M20.8* | AAATGAACCGTGGACACACA | ACCACCCTGAATCACAAAGC |
| *RP11-1020M18.10* | GCTGTCACATTGGATGGAAG | TTCTCCTGGCTTGCAAAGAT |
| *H19* | ACGAGGCCAGGTCTCCAG | CCAGCCTAAGGTGTTCAGGA |
| *RP11-195B3.1* | CTCCCGGAGCTCAAGCAA | GTGGCTCAGTCCTGTTGTCC |
| *RP11-554I8.2* | CAGAACAGAGCCACCTCCAG | GCACCGTCTGTTGTTCATTC |
| *ZNF503-AS1* | TAACTGGTGAAGCCCGGAAG | TCGACTGGGTCATTAAATGCT |
| *ESRG* | AAACAAGGCCTCTGAGGTAGG | TGAATTCCAGGTTCCTCACA |
| *LINC00617* | TTTTCCTTCTCCTCGCCTTC | ATCTTCCTTGCCAGGCTAAC |
| *RP11-469A15.2* | CCCCATCCATCTCTTCATCT | CGATATGTGGTGATTCTCTTCTTC |
| *LINC01162* | CTTTATCACCTATGTGACTTTAGGAGT | AGCCAGTACCATGGGTAGGA |
| *LINC00173* | GCTCCAAAAGGAAAGATACCA | ATGTGGCACAGCCTGACC |
| *MITF* | AGCGTCCTGTATGCAGATGG | CCGAGACAGGCAACGTATTT |
| *SOX2* | CACAACTCGGAGATCAGCAA | GGGCAGCGTGTACTTATCCT |
| *POU5F1* | GCAAAACCCGGAGGAGTC | TGGCTGAATACCTTCCCAAA |
| *NANOG* | ACAGGTGAAGACCTGGTTCC | CTGAGGCCTTCTGCGTCAC |
| *RPE65* | TACAGAAAGCACTGAGTTGAGC | CCATTTAGTAAGTCCACATTCATTTCC |
| *RLBP1* | GCTGCTCAGAGGCTATGTGA | TGCCTGCAAGATCTCATCAA |
| *LRAT* | TCAGACCTACCAGTTCTGCAA | CAAACAGGGTCACCGACTG |
| *MERTK* | AGTGCAGGGATTTCCAAAGA | GGGGCATAATCTACCCAACC |
| *BEST1* | CCTGCTGAACGAGATGAACA | CCACAGTCACCACCTGTGTA |
| *KRT18* | GGAGACTTCAAACTCCAGGATG | TCTGTGCTTGAGGACACAAC |
| *TJP1* | CTTCCAGAACCAAAGCCTGT | ATGCTGGGCCGAAGAATC |
| *CTNNB1* | GGGATCAAATCTGACACCAAA | CCTCAGCTCCCTGGTCAAT |
| *ZNF503* | GAACTGGCCACATTTTGCAC | CCGATTTGGTGTCCTTGTCG |

| **Supplementary Table S3. Antibodies used in this study** | | | |
| --- | --- | --- | --- |
| **Anti-protein** | **Host** | **Dilution and Application** | **Supplier** |
| GAPDH | Rabbit | 1:5000, Immunoblotting | Bioworld |
| Mertk | Rabbit | 1:1000, Immunoblotting | Cell Signaling Technology |
| Cytokeratin-18 | Rabbit | 1:10000, Immunoblotting | Abcam |
| ZO-1 | Rabbit | 1:200, Immunoblotting; 1:100, Immunofluorescence | Invitrogen |
| β-catenin | Rabbit | 1:1000, Immunoblotting; 1:100, Immunofluorescence | Cell Signaling Technology |
| BEST1 | Mouse | 1:100, Immunofluorescence | Abcam |
| NF-κB p65 | Rabbit | 1:1000, Immunoblotting | Cell Signaling Technology |
